# A Novel Primary Cell Line Model of Localized Prostate Cancer and Radioresistance—A Role for Nicotinamide N-Methyltransferase

**DOI:** 10.3390/cells14110819

**Published:** 2025-05-31

**Authors:** Jessica A. Wright, Stephanie D. White, Gavin Frame, Ana Bosiljkov, Shahbaz Khan, Roni Haas, Qian Yang, Minzhi Sheng, Xiaoyong Huang, Geoff S. Higgins, Ian Mills, Michelle R. Downes, Danny Vesprini, Hans T. Chung, Robert A. Screaton, Hon S. Leong, Paul C. Boutros, Thomas Kislinger, Stanley K. Liu

**Affiliations:** 1 Sunnybrook Research Institute, Toronto, ON M4N 3M5, Canada; 2 Department of Medical Biophysics, University of Toronto, Toronto, ON M5G 2C4, Canada; 3 Princess Margaret Cancer Centre, University Health Network, Toronto, ON M5G 2M9, Canada; 4 Departments of Human Genetics and Urology, University of California, Los Angeles, CA 90095, USA; 5 Jonsson Comprehensive Cancer Center, University of California, Los Angeles, CA 90024, USA; 6 Department of Oncology, University of Oxford, Oxford OX3 7DQ, UK; 7 Nuffield Department of Surgical Sciences, University of Oxford, Oxford OX3 7DQ, UK; 8 Department of Anatomic Pathology, Sunnybrook Health Sciences Centre, Toronto, ON M4N 3M5, Canada; 9 Department of Laboratory Medicine and Pathobiology, University of Toronto, Ontario, ON M5S 3K3, Canada; 10 Department of Radiation Oncology, University of Toronto, Toronto, ON M5T 1P5, Canada; 11 Department of Biochemistry, University of Toronto, Toronto, ON M5G 1M1, Canada

**Keywords:** prostate cancer, preclinical models, cell culture, radiotherapy

## Abstract

Prostate cancer cell lines are particularly clinically homogenous, mostly representing metastatic states rather than localized disease. While there has been significant work in the development of additional models, few have been created without oncogenic transformation. We derived a primary prostate cancer cell line from a patient with localized Gleason 7 prostate cancer—designated CaB34—which spontaneously immortalized. We leveraged CaB34 to generate a paired radioresistant subline, CaB34-CF, using a clinically relevant fractionated radiotherapy schedule. These two paired cell lines were investigated extensively to determine their molecular characteristics and therapy responses. Both CaB34 and CaB34-CF express prostate-specific markers, including KRT18, NKX3.1, and AMACR. Multi-omic analyses using RNAseq and shotgun proteomics identified NNMT as the most significantly dysregulated component in CaB34-CF. A bioinformatic analysis determined that NNMT was more abundant within prostate tumors compared to benign prostate, suggesting a role in tumor progression. Knockdown studies of NNMT demonstrated significant radiosensitization of CaB34-CF cells, which was largely a result of increased radiation-induced cellular senescence. Growth as 3D organoids was significantly higher in the CaB34-CF line, and demonstrated a less structured pattern of expression of cytokeratin markers. Radiosensitization with NNMT siRNA was recapitulated in a 3D organoid clonogenic assay in CaB34-CF cells. Our research provides a paired primary model of treatment-naïve and radioresistant disease to address mechanisms of therapy resistance, while expanding the repertoire of localized prostate cancer cell lines for the research community. In addition, our findings present NNMT as a potential therapeutic target for sensitization of radioresistant disease.

## 1. Introduction

More than 1.4 million new cases of prostate cancer are diagnosed worldwide annually, and it is the fifth leading cause of cancer death among men [1]. Prostate cancer is usually diagnosed after an elevated prostate specific antigen (PSA) blood test result, leading to a prostate biopsy for pathological confirmation of cancer. A pathologist assigns a Gleason score/grade group based upon the observed patterns and degree of cellular differentiation. Staging investigations typically include a bone scan, cross-sectional imaging with computed tomography (CT), and magnetic resonance imaging (MRI). Collectively, this provides a risk group categorization for the patient’s prostate cancer (low, intermediate or high risk) that guides treatment options and patient prognosis. Approved treatment options include surgery or radiotherapy [2].

Despite a similar worldwide incidence to breast and lung cancers, there exists a disparity in the quantity and diversity of cell lines available. There are four cell lines commonly used for the majority of prostate cancer research (22RV1, DU145, LNCaP, PC-3). All were derived from men over 20 years ago, and three of the four lines were established using tissue from metastatic sites. Cancer cell lines are the backbone of pre-clinical research, in part due to their simplicity to culture, expand, manipulate, and share. The scientific influence of 2D cell line models cannot be overstated; cell lines are used extensively, from compound screening to develop novel therapies to genomic-level investigations of prostatic carcinogenesis. This small, non-diverse collection of prostate cancer cells is a significant barrier to accurate representation of patient heterogeneity, and should be used with caution for generalizations about our understanding of prostate cancer. A notable high-throughput drug screen panel, the NCI-60 human tumor cell line screen, was used to screen thousands of compounds for their anti-tumor potential, but it uses only two of the aforementioned metastatic prostate cancer cell lines. Strikingly, the Cancer Cell Line Encyclopedia (CCLE), which compiles over 940 human cancer cell lines, only includes eight prostate cancer cell lines, which include the three mentioned above [3]. Thus, prostate cancer cell lines represent less than 1% of the CCLE, despite it being the most common non-skin malignancy in men. The low quantity of clinically representative prostate cancer cell lines is a major issue in the current “omic” era, in which a limited number of lines are used to make broad conclusions about prostate cancer’s molecular characteristics.

Radiotherapy is one of the main treatment modalities for localized prostate cancer. However, for men with high-risk prostate cancer treated with radiotherapy, up to 50% will have biochemical recurrence within 10 years [4]. This recurrent disease, known as radiorecurrent prostate cancer, can be clinically challenging to salvage due to the frequent inability to reirradiate at a curative dose because of normal tissue toxicity [5]. Radiorecurrent prostate cancer can cause considerable morbidity and mortality [5]. This can include pain or bleeding from invasion of adjacent organs and structures, as well as metastatic dissemination to the lymph nodes, bones or visceral organs, which results in reduced patient survival. Typically, radiorecurrent disease is managed with androgen deprivation therapy. However, this is a non-curative approach. Due to the DNA-damaging nature of radiation therapy, radiorecurrent tumors have increased genomic instability, which may confer more aggressive and metastatic phenotypes [6]. There is a paucity of clinical material available on radiorecurrent disease and, thus, the establishment of radioresistant prostate cancer models is of great importance to elucidate the underlying biology and develop new therapies.

To address these issues, we established and characterized a spontaneously immortalized primary prostate cancer cell line, designated CaB34, derived from a patient of Black Caribbean background. In addition, we describe the generation of a radioresistant subline—CaB34-CF—from CaB34 following exposure to fractionated radiotherapy mimicking a clinical schedule, and we perform multi-omics profiling and characterization. Both cell lines have growth characteristics and propagation abilities similar to the current widely used models, but they are representative of primary disease, making them novel models that can easily be incorporated into prostate cancer research for better representation. Through multi-omics analyses, we discovered that nicotinamide N-methyltransferase (NNMT) was the most significantly enriched target (mRNA and protein) in CaB34-CF cells. Indeed, NNMT expression is elevated in a range of human cancers and implicated in driving tumor progression [7,8]. Furthermore, NNMT has been linked to both radiosensitivity and drug sensitivity [9,10,11,12]. We demonstrated that knockdown of NNMT results in significant radiosensitization of CaB34-CF cells, through increased cell senescence following irradiation. This implicates NNMT as a potential novel target for treatment of radioresistant prostate cancer. Together, our primary spontaneously immortalized prostate cancer cell line derived from localized disease and its paired radioresistant cell line provide new models to uncover molecular mechanisms of radioresistance, while expanding the heterogeneity of lines available to the research community.

## 2. Materials and Methods

### 2.1. Derivation of CaB34

Patient characteristics are summarized in Table 1. The patient presented with high-volume, Gleason Score 7 (3 + 4) (ISUP Grade Group 2), localized prostate cancer. Biopsy core samples were taken from the primary tumor site at the time of brachytherapy treatment in 2020 and were immediately transported to the laboratory. Biopsy cores were washed thrice with phosphate-buffered saline (PBS), and then one core was formalin-fixed for pathology, one core was used for derivation, and additional cores were cryopreserved. The derivation core was finely minced using a scalpel into ~1 mm pieces, and then placed in 0.5 mg/mL collagenase I solution (Worthington Biochemical) for overnight digestion using orbital shaking at 37 °C and 80 rpm. The following day, the biopsy core was further trypsin-digested for 30 min, which was subsequently inactivated and removed through several PBS washes. Cells were then plated on a collagen-coated 10 cm dish (Corning, Corning, NY, USA) in F-media (components detailed in Table 2) with inactivated STO cells, based on a protocol adapted from Liu et al. [13]. Fresh inactivated STO cells were generated by irradiating cells collected in a Falcon tube with 30 Gy using a Faxitron CP160 x-ray irradiator (Faxitron, Tucson, AZ, USA). The formalin-fixed core was stained with hematoxylin and eosin, and provided to an expert pathologist for review. The biopsy was confirmed to be Gleason Grade 2, with 95% core involvement.

### 2.2. Cell Culture

STO feeder cells were obtained from Dr Ian Mills (University of Oxford) and were grown in DMEM with 10% FBS and 1% P/S. DU145 (RRID:CVCL_0105), and LNCaP (RRID:CVCL_0395) cells were grown in DMEM and RPMI respectively, with 10% FBS and 1% P/S supplementation.

### 2.3. STR Analysis

FTA Sample Collection Kit for Human Cell Authentication (ATCC) was used according to manufacturer’s instructions and returned to ATCC for analysis.

### 2.4. Oncomine v3 Assay

Genomic DNA from cultured cells was extracted using QIAmp DNA mini kit (Qiagen, Redwood City, CA, USA). For FFPE biopsy cores, an H&E stained slide was examined by an experienced pathologist to identify areas of prostate cancer. The remaining FFPE block was microdissected and genomic DNA was extracted according to manufacturer’s instructions using the RecoverAll total nucleic acid isolation kit (Invitrogen, Waltham, MA, USA). DNA concentration and quality were assessed using the Qubit dsDNA High Sensitivity Assay Kit (Thermofisher Scientific, Waltham, MA, USA). DNA was sent to the Genomics Core Facility at Sunnybrook Research Institute for analysis using Oncomine Comprehensive Assay v3 (OCAv3; Thermofisher Scientific). The OCAv3 dataset has been submitted to SRA (BioProject: PRJNA1164709).

### 2.5. Generation of Radioresistant Cells

Adherent CaB34 cells were irradiated using a Faxitron CP160 x-ray irradiator. Cells received a total of 78 Gy, given in 39 fractions of 2 Gy per fraction (initially one fraction per week, then two fractions per week as tolerated by the cells). This schedule was chosen to mimic a clinical conventional fractionation radiotherapy schedule used for localized prostate cancer. After recovery, cells were assayed for clonogenic survival compared to non-irradiated parental CaB34 cells.

### 2.6. Clonogenic Survival

Cells were seeded in triplicate in 6-well plates and irradiated 18 h later with a 4, 6, or 8 Gy dose, or mock-irradiated as a 0 Gy control. Plates were incubated for 7–14 days to allow for colony formation. Cells were stained using crystal violet and surviving colonies were counted (defined as >50 cells). Surviving fraction of the irradiated cells was calculated relative to the plating efficiency of the 0 Gy control cells. Radiation dose response curves were generated by fitting the surviving fraction data to the linear quadratic formula S = e − αD-βD2, where S is the surviving fraction, α and β are inactivation constants, and D is the radiation dose in Gy. Mean, SEM, and statistical significance were graphed on a logarithmic scale.

### 2.7. Proliferation Assay

Cells were seeded in triplicate in 6-well plates at a concentration of 3 × 10^4^ and 1.5 × 10^4^ and irradiated 18 h later with a 6 Gy dose or mock-irradiated with a 0 Gy control, respectively. Plates were incubated for 3–6 days to allow for cell growth. Once cells neared confluency, they were counted using trypan blue exclusion with the Countess automated cell counter (Invitrogen). Results are expressed as the fold-change in the number of cells from seeding to counting, normalized to the parental control at each dose. Three biological replicates were performed.

### 2.8. Senescence Assay

Cells were seeded in 6-well plates and irradiated 18 h later with a 6 Gy dose and a mock-irradiated 0 Gy control. Plates were incubated for 3–6 days. Cells were fixed and stained using a Senescence beta-Galactosidase Staining Kit (Cell Signaling Technologies 9860S, Danvers, MA, USA). The number of senescent cells was assessed using minimum of 5 distinct image sections of the well and averaged. Three biological replicates were performed.

### 2.9. Cell Cycle Analysis

Cells were seeded in 6-well plates and irradiated 18 h later with 0 Gy mock irradiation and 6 Gy doses for collection at 24 h post-irradiation. During collection, cells were isolated in 50 µL of a PBS/FBS solution, fixed using 80% ethanol, and stored at 4°. After both collection times were processed, the cells were prepared for FACS. Storage solution was aspirated, and cells were stained in propidium iodine (0.1 mg/mL; Sigma-Aldrich) with 0.6% NP-40 (Thermo Fisher Scientific) and RNAse A solution (RNAse A in HBSS) for 30 min in the dark on ice. Cells were strained and kept on ice until processing. A Symphony A3 Flow Cytometer was used to capture 20,000 events and subsequent analysis was performed using FlowJo software (Version 10.9.0 FlowJo LLC, Ashland, OR, USA). Three biological replicates were performed.

### 2.10. Soft Agar Assay

The bottom layer was made in quadruplicates using 2XDMEM/F12, 0.7% Agar, FBS, and P/S and allowed to cool for 1 h at room temperature. The cell layer was made using 2XDMEM/F12, 0.5% Agar, FBS, P/S, and 700 cells and put atop the bottom layer. A total of 300 µL of culture media was put atop the cell layer and replaced as needed over the course of the experiment. Anchorage-independent colony formation was assessed and counted after 4 weeks. Three biological replicates were performed.

### 2.11. Western Blot

Cells were lysed in cold RIPA buffer (0.5% sodium deoxycholate, 0.1% SDS, 1% NP-40), sonicated, and cold-centrifuged to remove cell debris. Protein was quantified using Bradford assay, and 20 µg of cell lysate was loaded onto 4–20% gradient gels (Bio-Rad, Hercules, CA, USA). After wet transfer onto polyvinylidene fluoride membranes, non-specific binding was blocked with 5% milk, and then membranes were incubated in primary antibody overnight. See Supplementary Table S1 for primary antibody information. The following day, membranes were washed, and then incubated with 1:5000 HRP-linked secondary anti-rabbit (Cell Signaling 7074) or anti-mouse (Cell Signaling 7076) antibody, as specified for each primary antibody. Protein signal was detected using Clarity Western ECL substrate (Bio-Rad) and ChemiDoc Imaging System (Bio-Rad). Western blot experiments performed in three biological replicates. Full membrane images available in Supplementary File S1.

### 2.12. qRT-PCR

Total RNA was extracted using the RNeasy kit (Invitrogen) and evaluated using a Nanodrop 2000 spectrophotometer. In total, 2000 ng of RNA with 260/280 and 260/230 > 1.9 was reverse-transcribed to cDNA using Vilo SuperScript cDNA Synthesis kit (ThermoFisher Scientific). Quantitative RT-PCR was performed on a QuantStudio 3 machine using TaqMan Fast Advanced Master Mix, according to manufacturer’s directions (ThermoFisher Scientific). All primers were obtained from ThermoFisher Scientific; see Supplementary Table S2 for catalog numbers. Biological triplicate data were normalized to CaB34 replicate #1 using the 2^(−ΔΔCt)^ method to obtain relative quantification (RQ). Heatmap of log2 transformed RQ data was generated using Morpheus by Broad Institute (RRID:SCR_017386).

### 2.13. mRNA-Seq

Total RNA was extracted using the Qiagen RNeasy Mini kit (Cat. No. 74104). RNA was submitted to Novogene (University of California Davis), where mRNA library preparation and sample quality control were undertaken according to their outlined procedures. mRNA-seq was performed using llumina NovaSeq 6000 with 150 bp paired-end reads. On average, approximately 58.5 million clean reads were generated for each sample. Reads were mapped to GRCh38/hg38 reference genome using HISAT2 v2.0.5 (RRID:SCR_015530). Gene expression was quantified using featureCounts v1.5.0-p3 [14] (RRID:SCR_012919), and differential gene analysis performed using the DESeq2R package (1.20.0) [15], with differential expression defined as |log2(FoldChange)| >= 1 & p-adj <= 0.05. The datasets generated during the current study are available in the GEO repository (GSE277693).

### 2.14. Proteomic Sample Preparation and Shotgun Proteomics

The cell pellets were resuspended in 200 μL of lysis buffer (50 mM HEPES pH 8, 1% SDS) followed by repeated freeze–thaw cycles, sonication, and subsequent processing, as previously described [16]. Magnetic-bead-based SP3 protocol [17] was used to capture proteins prior to digestion.

Mass spectrometry was performed as previously described [16], with raw data processed using MaxQuant [18] (RRID:SCR_014485) version 1.6.3.3, searching against a UniProt protein sequence database containing human protein sequences from Uniprot (complete human proteome; 2022-04). LFQ intensities were utilized for protein quantitation, with median-adjusted iBAQ values used as replacements for proteins with missing LFQ values [19]. Protein intensities were log2-transformed for subsequent analysis. The datasets generated are available in the MassIVE repository (MSV000095893).

### 2.15. siRNA Knockdown

Knockdown of NNMT was performed using siTran siRNA transfection (Origene, Rockville, MD, USA) with NNMT siRNA (Origene—SR303197) according to manufacturer’s instructions. Knockdown was confirmed using qRT-PCR and Western blotting.

### 2.16. Mito Stress Assay

Measurement of the metabolic rate of the CaB34 and CaB34-CF was performed in vitro, as previously described [20]. Oxygen consumption rate profiles of the cells were measured (*n* = 8 wells per group) using a live cell metabolic assay platform (Seahorse XF analyzer with Cell Mito Stress Test kit, Agilent, Santa Clara, CA, USA) in the presence of 10 mM glucose, 1 mM pyruvate, and 2 mM glutamine. This allowed the measurement of the oxygen consumption rate due to ATP production, the proton leak, and the maximal respiration and non-mitochondrial oxygen consumption rate, respectively, so that relative metabolic rate could be compared between parental and radioresistant cells.

### 2.17. Organoid 3D Droplets

Cells were collected in suspension in F-media, and then combined with growth factor reduced Matrigel (Corning) to required cell density. Cell and media volume was limited to below 10% of final Matrigel mixture volume. Droplets were plated and immediately inverted for 15 min at 37 °C to solidify, and then wells filled with appropriate volume of F-media. For non-irradiated organoid formation assays, 250 cells in 10 μL droplets were seeded in triplicate. For organoid clonogenic assays, cell density was increased to 500 cells per 10 ul droplets for the 6 Gy condition, and droplets were irradiated 24 h after seeding. On Day 7, 4X images were captured of droplets with an inverted microscope using LC Micro software (version 2.4), and then organoids were quantified and measured using ImageJ (version 1.54g). A minimum area of >2000 μm^2^ was set as the threshold for “formed” organoids, while the circularity constraint was set to >0.2.

### 2.18. Organoid Immunofluorescence Imaging

The organoids were extracted from Matrigel using Cell Recovery Solution (Corning, Somerville, MA, USA). Following the depolymerization of Matrigel, the free-floating 3D cultures were fixed in 10% formalin and permeabilized with 0.5% Triton X-100. Following permeabilization, the organoids were incubated in a blocking buffer (5% BSA, 0.1% Triton X-100, 0.05% Tween-20) for 1 h at room temperature with gentle rocking. Incubation with primary antibodies was carried out overnight (KRT5 (Cell Signaling 25807—anti-Rabbit) and KRT8 (Biolegend 904804—anti-Mouse)). After washing, organoids were incubated with Cyanine3 Donkey anti-rabbit IgG (Biolegend 406402) and Alexa Fluor 488 Goat anti-mouse IgG (Biolegend 405319) secondary antibodies at room temperature, and then nuclear-stained with DAPI. Organoids were then subjected to optical clearing with an 88% glycerol solution for 20 min at room temperature, and images acquired using confocal microscopy (Nikon A1R MP).

### 2.19. Statistical Analysis

Statistical analysis was performed using GraphPad PRISM 10.2.3 (GraphPad Software, San Diego, CA, USA). Mean values between two groups were tested for significant differences using two-tailed Student’s *t*-tests. Means of groups of 3 or more were compared using 1-way ANOVA with Bonferroni’s multiple comparison correction. Data graphed as mean ± SEM unless otherwise stated. * *p* < 0.05; ** *p* < 0.01; *** *p* < 0.005; **** *p* < 0.0001. All significant comparisons shown on graphs; otherwise, results are non-significant.

## 3. Results

### 3.1. Cell Line Derivation

**Figure 1:**
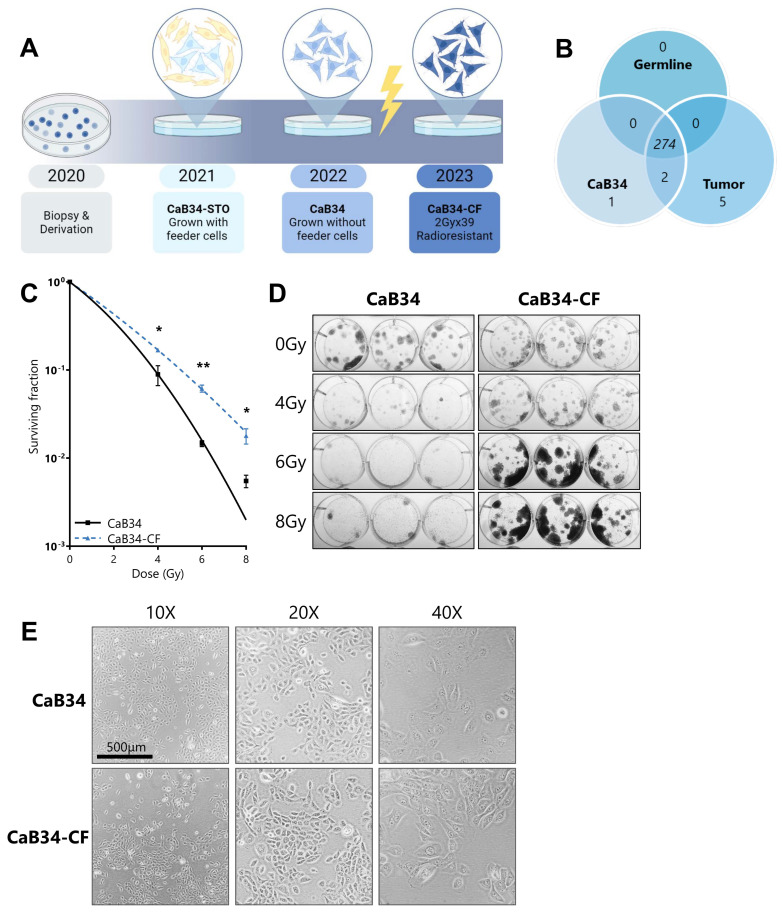
Derivation and characterization of CaB34 and sublines. (**A**): Timeline of CaB34 culture and subline relationships. (**B**): Overlap in DNA alteration results from Oncomine Comprehensive Assay v3 with patient germline DNA, patient tumor DNA, and CaB34 DNA. (**C**): Clonogenic survival curves of CaB34 and CaB34-CF. Surviving fraction data are fitted to the linear quadratic equation with mean and SEM plotted, *n* = 3. (**D**): Representative images of crystal violet-stained clonogenic assay plates illustrating pronounced differences between CaB34 and CaB34-CF clonogenic survival. (**E**): Representative microscopy images of CaB34 and CaB34-CF cell lines at 10×, 20×, and 40×. Gy = Gray; * = *p* < 0.05; ** = *p* < 0.01.

A 71-year-old patient of Black Caribbean descent consented to have research biopsies taken from his Gl7 (3 + 4) prostate cancer. Dissociated prostate biopsy cells were plated in FM with inactivated murine STO feeder cells on a collagen-coated dish (see Materials and Methods for detailed procedures). Epithelial cell colonies were observed within 5 days, and feeder cells were added as required to maintain confluency. To test the effect of media formulation on indefinite proliferation, epithelial cells with FM and STO cells were continuously maintained for 86 passages over 18 months (denoted CaB34-STO). To facilitate their characterization, pre-clinical evaluation, and ease of experimentation, we evaluated the ability of CaB34-STO to grow without feeder cells. This feeder-free cell line (designated CaB34) thrived and was grown as a monolayer for over 200 passages without undergoing terminal growth arrest (see Figure 1A for culture timeline).

### 3.2. STR Profiling

To further establish CaB34 as a unique cell line, STR profiling was performed. The CaB34 cell line was authenticated as human and did not match any known cell lines in the ATCC or Expasy STR databases (Table 3). Furthermore, we confirmed the absence of STO mouse feeder cells via qRT-PCR (Supplementary Figure S1).

### 3.3. Genomic Alterations

Oncomine Comprehensive Assay v3 (OCAv3) next-generation sequencing was performed using DNA from three sources: patient germline DNA, patient tumor DNA, and CaB34 DNA. In total, 274 alterations were common between the germline, tumor, and CaB34, including single-nucleotide variants (SNVs), insertion/deletions, and multiple-nucleotide variants (MNVs) (Figure 1B). There were no significant copy number variations (CNVs) detected across the 144 genes tested. All the alterations identified in the germline DNA were present in both tumor and CaB34. None of the variants were listed as pathogenic in ClinVar [21]. There were seven distinct SNVs present in tumor DNA, with two of these also detected in the CaB34—*NOTCH3* missense and *FANCD2* synonymous mutation.

### 3.4. Generation of Radioresistant Cell Line

CaB34-CF cells were generated using a clinical radiotherapy schedule (39 fractions of 2 Gy doses; Figure 1A). Following recovery, CaB34-CF cells were evaluated for radioresistance using the clonogenic assay. CaB34-CF cells showed significant radioresistance compared with CaB34 cells (Figure 1C,D). There were no pronounced morphological differences between CaB34 and Cab34-CF (Figure 1E). Both cell cultures were examined by an expert genitourinary pathologist (Dr M.R.D.) and confirmed as epithelioid with prostatic characteristics, notably, prominent nucleoli and large central nuclei.

### 3.5. Molecular Characterization of Cell Lines

**Figure 2:**
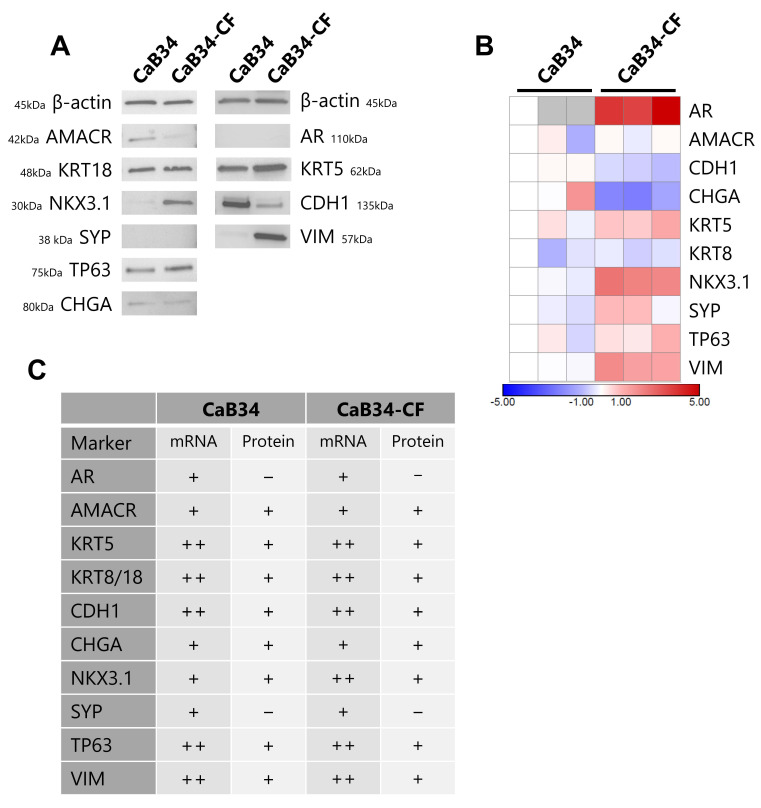
Molecular characterization of CaB34 and CaB34-CF. (**A**): Western blot data comparing protein expression between CaB34 and CaB34-CF. Representative images shown. (**B**): mRNA expression in CaB34 and CaB34-CF, as determined by qRT-PCR (*n* = 3). Heatmap displays Log2 transformed relative quantification data normalized to CaB34 biological replicate #1. Grey boxes indicate no detectable expression (Ct > 40). (**C**): Summary of Western blot and qRT-PCR data. ‘+’ indicates detection of the marker; ‘-’ specifies no detectable expression. For PCR data: + = Ct > 30; ++ =Ct < 30.

To evaluate the expression of epithelial and prostate markers in CaB34, we performed Western blotting analyses (Figure 2A). The prostate marker AR was not detected in either CaB34 or CaB34-CF cells, a common issue in the derivation of prostate cancer cell lines. The prostate luminal cell marker KRT18 was present in both cell lines, in addition to the basal cell markers KRT5 and TP63. AMACR and NKX3.1 were both positive for protein expression, which is expected with prostate adenocarcinoma. The neuroendocrine marker SYP was undetectable. These Western blot results confirm that the CaB34 and CaB34-CF cell lines are representative of prostate adenocarcinoma cells.

qRT-PCR was also performed to examine mRNA expression, and the results were largely concordant with the protein findings (Figure 2B). AR, KRT5, and SYP were detectable at the mRNA level, likely due to the increased sensitivity compared with Western blotting or post-translational modifications altering protein abundance. Interestingly, the expression of AR was distinctly higher in the CaB34-CF cell line. The qRT-PCR and Western blot results are summarized in Figure 2C.

### 3.6. Phenotypic Characterization of Cell Lines

**Figure 3:**
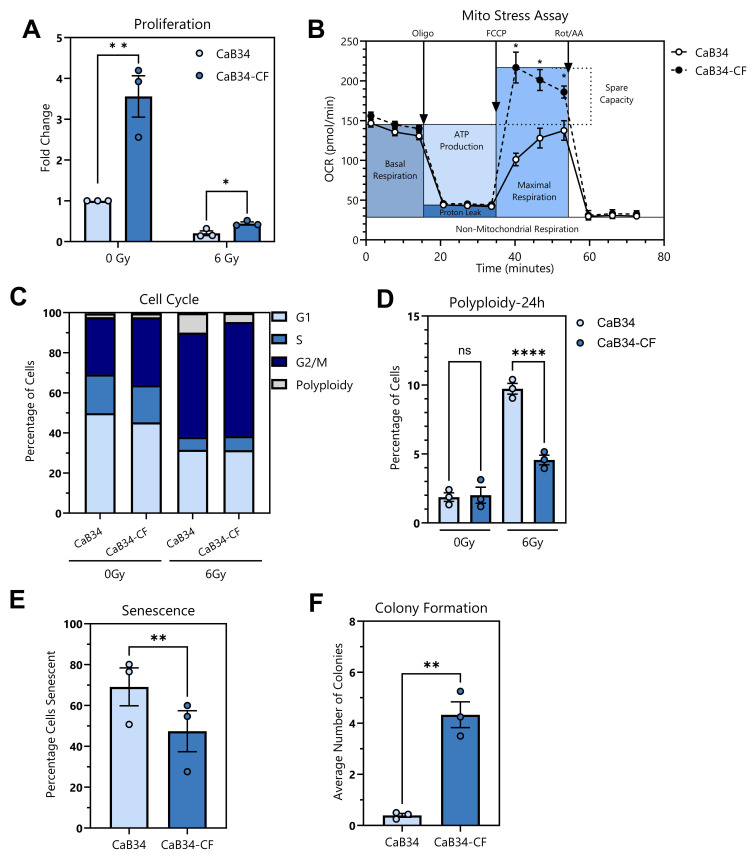
Phenotypic characterization of CaB34 and CaB34-CF. (**A**): Proliferation after mock irradiation (0 Gy) or 6 Gy irradiation. (**B**): Mito stress assay demonstrating oxygen consumption rate differences. (**C**): Cell cycle profiles after mock or 24 h post-6 Gy irradiation. (**D**): Polyploid population comparison from cell cycle analysis. (**E**): Percentage of cells senescent after 6 Gy irradiation. (**F**): Soft agar colony formation examining anchorage-independent growth capacity. Gy = Gray; * = *p* < 0.05; ** = *p* < 0.01; **** = *p* < 0.0001; ns = non-significant. OCR = oxygen consumption rate, Oligo = oligomycin, FCCP = p-trifluoromethoxy carbonyl cyanide phenylhydrazone, Rot/AA = rotenone and antimycin A.

CaB34-CF cells demonstrated a pronounced increase in baseline proliferation compared with CaB34 cells, and this significant effect was maintained after 6 Gy irradiation (Figure 3A). Using the Mito stress assay, the metabolic differences between CaB34 and CaB34-CF were examined. There were no basal respiration differences, but a significant increase in maximal respiration and spare capacity was observed in the CaB34-CF cells (Figure 3B). The cell cycle analysis demonstrated no significant difference in any phase of the cell cycle between CaB34 and CaB34-CF non-irradiated controls (Figure 3C, Supplementary Figure S2). This contrasts with the proliferation data, which clearly demonstrates an increase in the basal growth rate of the CaB34-CF cells. Additionally, there were no significant differences between the cell lines in G1, S, and G2/M phase fractions after 6 Gy irradiation. Interestingly, there was a significant decrease in polyploid cells in the CaB34-CF cell line after 6 Gy treatment, compared with CaB34 (Figure 3D).

Senescence after irradiation was examined to determine whether increased cell survival was responsible for radioresistance in the CaB34-CF population. After 6 Gy irradiation, CaB34-CF cells showed significantly reduced senescence compared to CaB34 cells (Figure 3E).

Finally, the soft agar assay (an in vitro surrogate for tumorigenicity) was performed. This revealed that the CaB34 cells had a minimal ability to grow in anchorage-independent conditions, whereas the CaB34-CF had a significantly enhanced capacity to form colonies (Figure 3F).

### 3.7. RNA Seq and Proteomic Analyses

**Figure 4:**
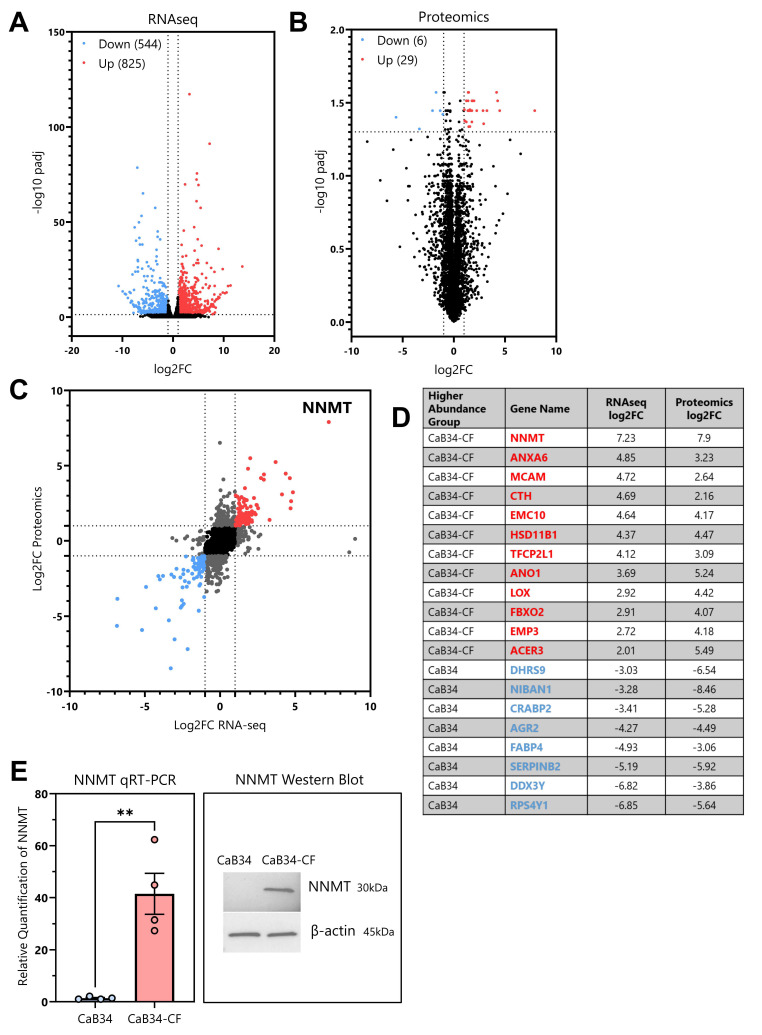
RNAseq and proteomic analyses of CaB34 and CaB34-CF cells. (**A**): RNAseq and (**B**): mass spectrometry proteomic comparison of CaB34-CF versus CaB34 cells (*n* = 3). Both graphed as log2FC versus –log10 adjusted *p*-value. Red dots (upper right) and blue dots (upper left) indicate significant up- or down-regulation, respectively, in CaB34-CF versus CaB34. (**C**): Combination of log2FC data from RNAseq and proteomics, highlighting targets with concordant changes in both datasets. (**D**): A selection of targets with the most pronounced up- or down-regulation in both datasets. (**E**): Confirmation of NNMT down-regulation at both mRNA and protein levels in CaB34-CF cell line using qRT-PCR and Western blotting. ** = *p* < 0.01.

RNAseq was performed to evaluate transcriptomic changes between CaB34 and CaB34-CF. The biological triplicates for the RNAseq were shown to have a strong correlation between samples (Supplementary Figure S3). There were 1369 significant differentially expressed genes between CaB34 and CaB34-CF (Figure 4A). In parallel, a mass spectrometry shotgun proteomics analysis was performed on CaB34 and CaB34-CF cellular lysates, which showed correlation between the biological replicates (Supplementary Figure S3). There were 35 significant differentially abundant proteins, with 29 enriched in CaB34-CF and six showing reduced abundance (Figure 4B).

Combining the RNAseq and proteomic data demonstrated a strong correlation between the log2FC datasets (Figure 4C) (r = 0.635, *p* < 0.0001). The targets with the most pronounced log2FC changes in both datasets are summarized in Figure 4D. We focused on NNMT for further investigation as a potential mediator of radiation resistance, as it had the greatest differential abundance at both the RNA and the protein level.

### 3.8. NNMT

**Figure 5:**
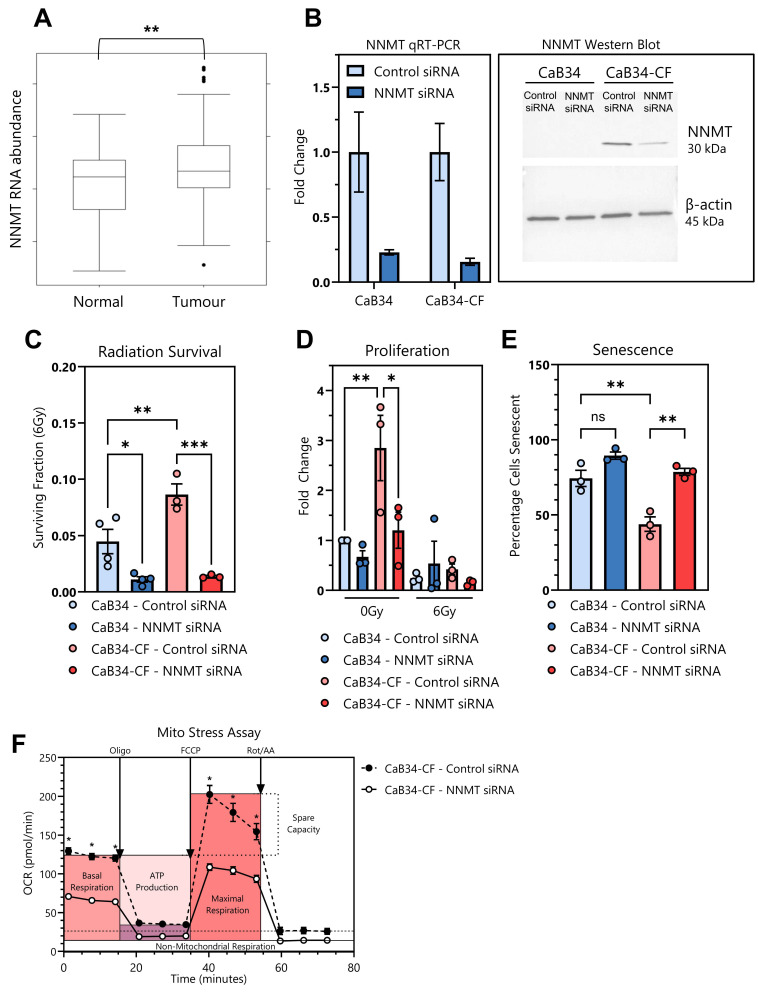
Effects of NNMT knockdown on radioresistant phenotype. (**A**): Data from TCGA showing elevated NNMT RNA expression in prostate cancer tumors, compared with normal prostate samples. *n* = 534; *p* = 0.002. (**B**): NNMT siRNA knockdown confirmation at the RNA and protein level by qRT-PCR (left panel) and Western blot (right panel). (**C**): Surviving fraction of CaB34 and CaB34-CF cells at 6 Gy from clonogenic assay with control or NNMT siRNA knockdown (*n* = 3). (**D**): Proliferation of cell lines after control or NNMT knockdown and mock (0 Gy) or 6 Gy irradiation. (**E**): Senescence of cell lines after control or NNMT siRNA knockdown with 6 Gy irradiation. (**F**): Mito stress assay of CaB34-CF cells with control or NNMT siRNA knockdown showing oxygen consumption over time. * = *p* < 0.05; ** = *p* < 0.01; *** = *p* < 0.001, ns = non-significant. TCGA = The Cancer Genome Atlas. OCR = oxygen consumption rate, Oligo = oligomycin, FCCP = p-trifluoromethoxy carbonyl cyanide phenylhydrazone, Rot/AA = rotenone and antimycin A.

We confirmed an increased NNMT abundance in the CaB34-CF cell line using qRT-PCR and Western blotting (Figure 4E). The evaluation of the tissue RNA from the TCGA showed that NNMT is in higher abundance within prostate tumors versus normal prostate (Figure 5A), as has been documented in other cancer types [8]. To evaluate a potential role for NNMT in radiation response, we performed siRNA knockdown of NNMT (Figure 5B). The knockdown of NNMT resulted in significant radiosensitization of CaB34-CF (Figure 5C). Interestingly, we also observed that NNMT knockdown reduced the surviving fraction in CaB34 cells, although this occurred to a lesser degree. To investigate the contribution of proliferation or senescence to NNMT radiosensitization, we performed proliferation and senescence assays. Unirradiated control siRNA-transfected CaB34-CF cells were significantly more proliferative than CaB34 cells, while NNMT knockdown significantly reduced the proliferation of CaB34-CF near to the parental CaB34 level (Figure 5D). Following irradiation, there was a decrease in proliferation observed in CaB34-CF cells with the NNMT knockdown, although statistical significance was not reached (Figure 5E). NNMT knockdown significantly increased the radiation-induced senescence of CaB34-CF cells to a level similar to that of the CaB34 parental cells (Figure 5E). This indicated that the radiosensitization of CaB34-CF cells following NNMT knockdown relies upon the induction of senescence. Due to its role in cellular metabolism, the effect of NNMT knockdown on oxygen consumption in CaB34-CF cells was examined. The NNMT siRNA significantly reduced the maximal respiration of CaB34-CF cells (Figure 5F) to a level similar to that of the CaB34 parental cells (Figure 3B). However, NNMT knockdown also significantly reduced the basal respiration rate of the CaB34-CF cells, a difference not observed between the parental and CaB34-CF cell lines. This suggests there may be additional factors contributing to metabolic differences in radiation-resistant CaB34-CF cells, and the effect is likely not to be mediated solely by NNMT up-regulation.

### 3.9. Organoid Formation

**Figure 6:**
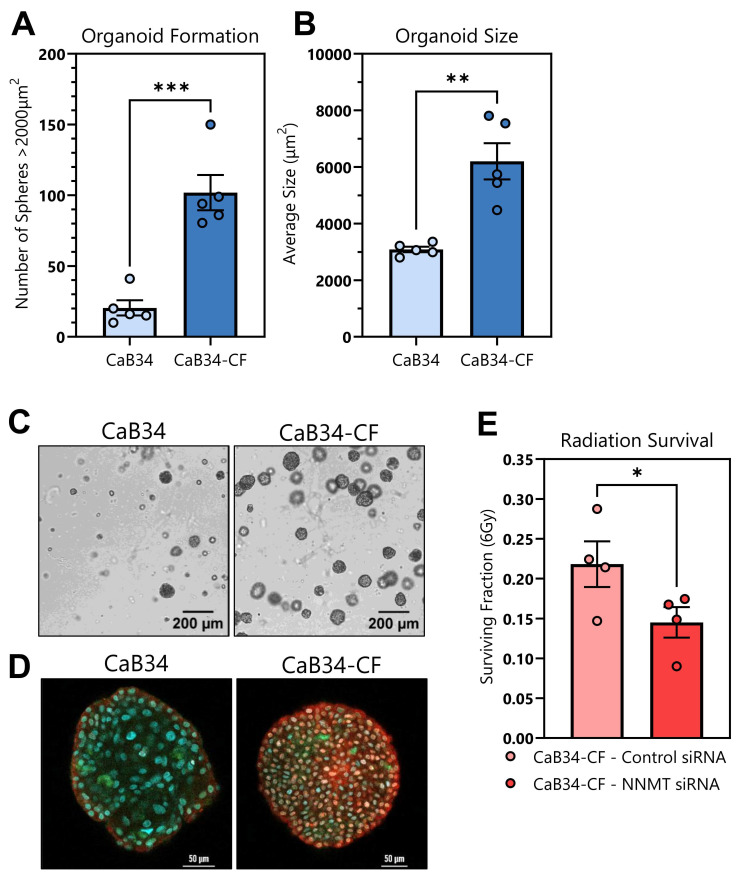
Organoid formation efficiency and effect of NNMT knockdown. (**A**): Comparison of quantity and (**B**): average size of organoids formed on Day 7 between CaB34 and CaB34-CF cells (formation threshold set as >2000 µm^2^). (**C**): Representative microscopy images of CaB34 and CaB34-CF organoids on Day 7. Scale bar = 200 μm. (**D**): Immunofluorescence image of a representative organoid from each cell line. Red = KRT5; green = KRT8; blue = DAPI. Scale bar = 50 μm. (**E**): Surviving fraction of organoid CaB34-CF cultures with control or NNMT siRNA knockdown following 6 Gy irradiation. * = *p* < 0.05; ** = *p* < 0.01; *** = *p* < 0.001.

To further evaluate the tumorigenic phenotypes of the CaB34 cell lines, they were grown as organoids in a 3D matrix for the purpose of better recapitulating the structural heterogeneity of prostate cancer tissue. As expected, the CaB34-CF cells formed organoids at a significantly higher level of efficiency than parental CaB34 (Figure 6A). In addition, the average size of CaB34-CF organoids was much larger than that of the CaB34 organoids (Figure 6B), consistent with their higher anchorage-independent soft agar colony formation, previously shown in Figure 3F. The differences in formation and size were visually very striking, as evident in Figure 6C. To assess differences in the composition of the organoids, immunofluorescence was performed. CaB34 organoids appeared to have organized expression patterns, with KRT5 stronger around the borders of the organoid (Figure 6D). Interestingly, the CaB34-CF organoids demonstrated more diffuse, non-polarized expression of KRT5 and KRT8 throughout the structure (Figure 6D).

The effect of NNMT knockdown on clonogenic survival in organoids was evaluated to validate the radiosensitization observed in 2D culture (Figure 5B). Reduction in NNMT significantly reduced the surviving fraction of CaB34-CF cells in a 3D clonogenic assay (Figure 6E), recapitulating the results observed in 2D (Figure 5B).

## 4. Discussion

The limited selection of prostate cancer cell lines currently used in pre-clinical studies were generated decades ago, mainly from biopsies of metastatic sites in patients. Amongst other factors, this is partially because cells derived from primary prostate tumors have proven to be notoriously difficult to establish in culture, compared with other cancer types. However, since these original cell lines were derived, technological advances and novel media formulas have allowed for further attempts to be made at expanding the currently inadequate compilation of prostate cancer cell lines.

Here, we characterize a spontaneously immortalized primary prostate cancer cell line derived from a patient of Black Caribbean descent, who presented with localized Gleason 7 (3 + 4) disease. This cell line—designated CaB34—grows rapidly in a healthy monolayer, and continued to proliferate for over 200 passages. Due to the growth and resilience of this novel model, it is an ideal candidate for integration into existing pre-clinical laboratory experiments, and we strive to promote collaboration with additional prostate cancer research groups to increase the diversity of pre-clinical models.

Previously, our laboratory generated radioresistant sublines from classical prostate cancer cell lines (DU145, PC3, 22RV1) [22,23,24,25]. However, the limitations associated with these classic cell lines persist in their respective sublines, and they thereby fail to be truly representative of the patient population receiving radiotherapy. Thus, after we established the CaB34 cell line, we generated a paired radioresistant cell line—designated CaB34-CF—through fractionated irradiation of the parental cell line. CaB34-CF demonstrates significant radioresistance and an aggressive phenotype characteristic of clinical radiorecurrent disease, resulting from pronounced increased baseline proliferation and reduced radiation-induced senescence, compared with the CaB34 cells. From the multi-omics data, we identified nicotinamide N-methyltransferase (*NNMT*) as the target with the strongest enrichment at both RNA and protein levels in the CaB34-CF radioresistant model. This suggested its possible role in the radiation resistance phenotype, which was further investigated through functional studies. NNMT is a SAM-dependent enzyme that methylates nicotinamide and is thus strongly involved in metabolic pathways and regulation. This led us to evaluate the metabolic differences between CaB34 and CaB34-CF, using the Mito stress assay. There was a strong increase in the maximal respiration of the CaB34-CF cells, which could have been due to increased NNMT in that population.

In response to NNMT knockdown, the CaB34-CF cells experienced greater radiosensitization and senescence compared to CaB34, suggesting that NNMT may be a driver of radioresistance. NNMT may influence radiation response through several different mechanisms. Firstly, NNMT reduces methylation potential in cancer cells by altering the SAM:SAH ratio [26]. This has widespread consequences for gene expression and response to cancer therapies, as previously reported [27]. It has also been hypothesized that elevated levels of NNMT may promote cancer radioresistance by removing the inhibitory block of nicotinamide upon PARP-1, thereby enhancing DNA repair [9,28]. This could contribute to the differences in radiation survival and polyploidy seen between the parental CaB34 and radiation-resistant CaB34-CF cells.

NNMT is enriched in a range of different human tumor types and may be important for tumor progression [8]. In agreement with this, through a bioinformatic analysis of TCGA, we discovered that NNMT was in greater abundance at the RNA level in prostate cancer compared to benign prostate tissue. Additionally, Zhou and colleagues reported that NNMT was up-regulated in prostate cancer specimens by immunohistochemistry, and negatively correlated with Gleason score [29]. Interestingly, they noted that elevated NNMT expression was correlated with improved outcomes in men with advanced prostate cancer. They postulated that the underlying mechanisms of NNMT in tumor progression may be distinct between primary and metastatic tumors. It remains to be determined whether NNMT has predictive or prognostic biomarker potential for prostate cancer patients with radioresistant cancer.

The utility of our paired primary derived CaB34 and radioresistant CaB34-CF cells for investigating mechanisms of radioresistance and cancer aggression is further highlighted by our multi-omics analyses (Figure 4D). We identified a panel of potential targets in addition to NNMT, which are of great interest for future investigation. These include Annexin A6 (*ANXA6*) and CD146/Melanoma cell adhesion molecule (*MCAM*), which were reported to promote radioresistance in nasopharyngeal carcinoma [30] and glioblastoma multiforme [31], respectively. Anoctamin 1 (*ANO1*) was implicated in maintaining stemness and invasive potential in glioma cancer stem cells [32]. Lysyl oxidase (*LOX*) was shown to mediate hypoxia-induced metastasis [33] and is secreted in a dose-dependent manner in response to ionizing radiation in a range of cancer cell types [34]. Alkaline ceramidase 3 (*ACER3*) was reported to promote proliferation and reduced cell death in hepatocellular carcinoma cells and was inversely correlated with survival in patients [35]. We believe that our CaB34-CF cell line models locally recurrent prostate cancer (since they were derived from primary localized prostate cancer cells (CaB34)), and support NNMT as a promising target to sensitize radioresistant prostate cancer cells. We believe that future research should investigate the use of small-molecule inhibitors of NNMT as potential therapeutics for overcoming prostate cancer radiorecurrence [36,37,38]. Future studies should also consider the impact of NNMT overexpression on radiation response.

We acknowledge the limitations of this research, as we were unable to grow these cells as orthotopic or subcutaneous tumor xenografts, and thus cannot account for microenvironmental influences on radiosensitivity. In order to address this limitation, we sought to validate the radiation survival impact of NNMT using 3D organoid assays, an emerging in vitro method for better recapitulating tumor characteristics and heterogeneity compared with traditional 2D culture. First, the organoid formation of the CaB34-CF radiation-resistant subline, compared with the parental, was markedly increased (Figure 6A); this is consistent with the increased soft agar colony formation seen using the classical soft agar tumorigenicity assay (Figure 3F). Further, the average size differences between the CaB34 and CaB34-CF organoids were congruent with the significantly higher proliferation of the CaB34-CF line compared with the parental CaB34 in 2D culture (Figure 3A). The consistency observed between our 2D and 3D culture results supports the continued utility of 2D culture for ease and reproducibility, while demonstrating the benefit of 3D organoids for unique observations that may not manifest in 2D. For example, the distinct pattern of cytokeratin expression in the CaB34 parental organoids is similar to the luminal/basal cell pattern which is observed in non-malignant prostate tissue (Figure 6D) [39]. The loss of this patterning in CaB34-CF organoids may suggest that these cells are a more de-differentiated population, with potential for future research seeking to address the effect of this organoid growth pattern on gene regulation and expression.

The low organoid formation ability of CaB34 posed a challenge for assaying survival following 6 Gy irradiation, and we were unable to reliably quantify the surviving fraction of this population to determine the effect of the NNMT knockdown. The CaB34-CF cell line showed significant radiosensitivity with NNMT reduction, which recapitulates the effect observed in 2D, and further supports the role of NNMT in radioresistance.

## 5. Conclusions

In conclusion, our CaB34 and CaB34-CF cells provide a clinically relevant model that offers insight into localized prostate cancer radiation response and aggression, in contrast to the classical prostate cancer cell lines currently utilized in research, which are derived from metastatic disease. NNMT was found to have a significant role in radioresistance, which has implications for future studies targeting radiorecurrent prostate cancer. Our vision is to establish a panel of primary derived prostate cancer cell lines from a diverse range of patients with localized disease, which will expand the repertoire of cell line models to comprehensively address mechanisms of therapy resistance and aggression.

## Figures and Tables

**Table 1:** Patient demographics. PSA = prostate-specific antigen. TNM = tumor, lymph node, metastasis. HDR BT = high dose rate brachytherapy. EBRT = external beam radiotherapy.

	Age	Ethnicity	ISUP Grade Group	PSA (ng/mL)	TNM	Risk Group	Hormone Therapy	Radiotherapy
CaB34	71	Caribbean	2	31.3	T1cN0M0	High	Yes —6 days prior to biopsy	15 Gy/1 HDR BT then 46 Gy/23 EBRT

**Table 2:** F-media components.

Component	Concentration in Media	Company	Catalog No.
3:1 *v*/*v* F-12:DMEM	-	Gibco, Waltham, MA, USA	
FBS	5%	Gibco	A3160701
Penicillin–Streptomycin	1%	Gibco	15140122
Adenine	24 µg/mL	Sigma-Aldrich (St. Louis, MO, USA)	A2786
Cholera Toxin	8.4 ng/mL	Sigma-Aldrich	C8052
EGF	10 ng/mL	Peprotech (Cranbury, NJ, USA)	AF-100-15
Hydrocortisone	0.4 µg/mL	Sigma-Aldrich	H0888
Insulin	5 µg/mL	Sigma-Aldrich	I9278
Y-27632	10 µM	StemCell Technologies (Vancouver, BC, Canada)	72307

**Table 3:** STR profiling results from selected alleles.

	D5S818	D13S317	D7S820	D16S539	vWA	TH01	AMEL	TPOX	CSF1PO
CaB34	8, 12	13, 14	8, 10	10, 12	16, 17	5, 7	X, Y	6, 11	11

## Data Availability

Proteomics datasets are available in the MassIVE repository (MSV000095893). RNA-seq datasets have been uploaded to GEO repository (GSE277693). Oncomine Comprehensive V3 assay data have been uploaded to SRA database (BioProject: PRJNA1164709).

## Supplementary Materials

The following supporting information can be downloaded at https://www.mdpi.com/article/10.3390/cells14110819/s1. Supplementary Figure S1: Raw Ct value plot of qRT-PCR data of human and mouse GAPDH in cell lines (STO = mouse fibroblasts; LNCaP, DU145 = human prostate cancer). Supplementary Figure S2: Complete cell cycle analysis for CaB34 and CaB34-CF. One-way ANOVA with Bonferroni’s multiple comparison correction. * *p* < 0.05; ** *p* < 0.01; *** *p* < 0.005; **** *p* < 0.0001, ns=non-significant. Supplementary Figure S3: Heatmap clustering of CaB34 and CaB34-CF (*n*=3 biological replicates) for RNAseq R2 Pearson correlation (upper panel) and proteomic samples (lower panel). Supplementary Table S1: Western blot antibodies. CST = Cell Signaling Technology. MW = molecular weight. Supplementary Table S2: Taqman qRT-PCR primers. Supplementary File S1: Full Western Blot Membrane Images.
