# Supplementary material for: A Novel Primary Cell Line Model of Localized Prostate Cancer and Radioresistance—A Role for Nicotinamide N-Methyltransferase
Source: Cells. 2025 May 31;14(11):819. doi: 10.3390/cells14110819 (PMC12153919; doi:10.3390/cells14110819)
Supplement: Supplementary file 1 [file cells-14-00819-s001.zip › Supplementary File 1.pptx]

## Slide 1
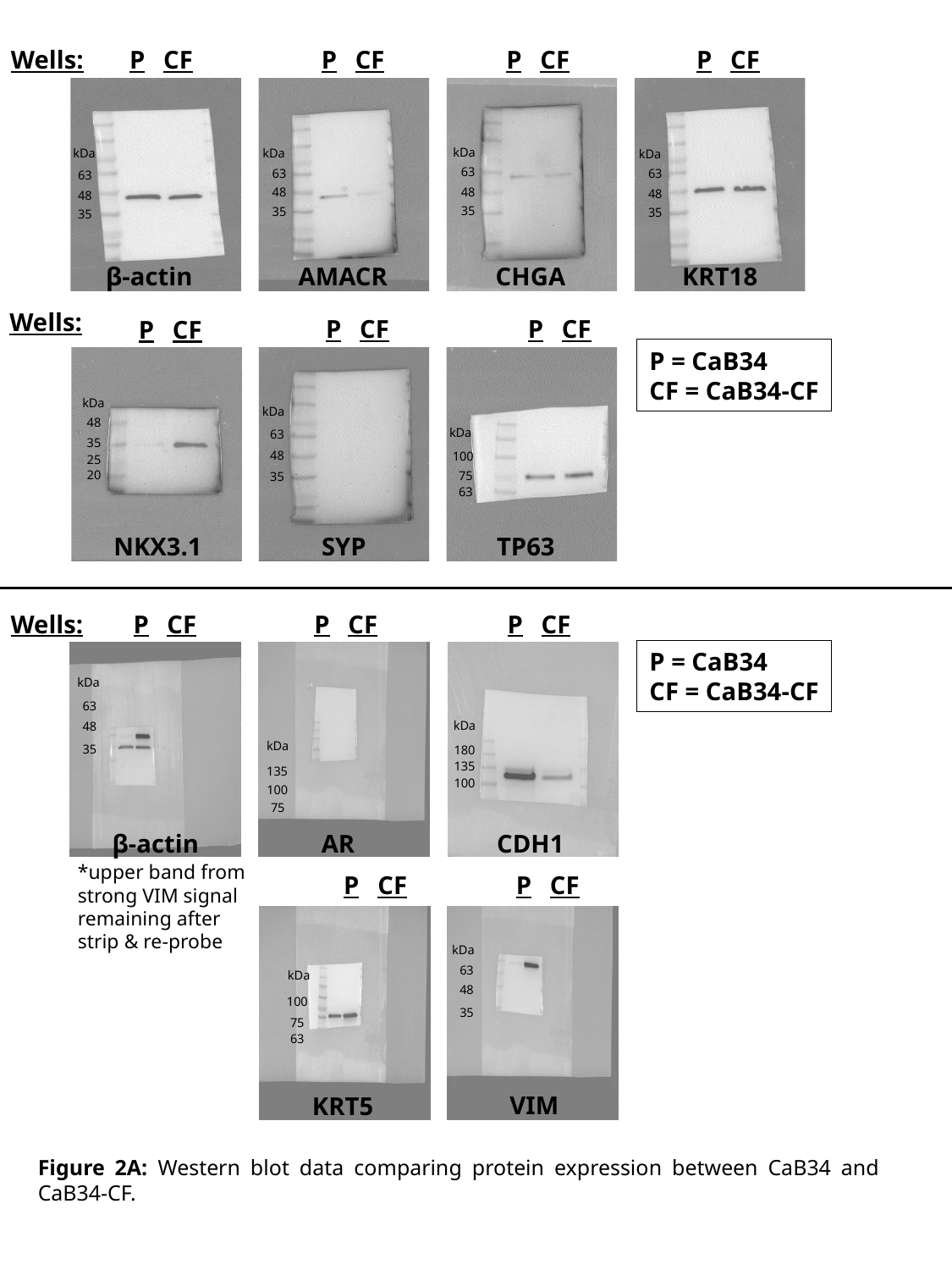

Wells:
P
CF
P
CF
P
CF
P
CF
kDa
kDa
kDa
kDa
63
63
63
63
48
48
48
48
35
35
35
35
β-actin
AMACR
CHGA
KRT18
Wells:
P
CF
P
CF
P
CF
P = CaB34
CF = CaB34-CF
kDa
kDa
48
kDa
63
35
48
100
25
20
75
35
63
SYP
TP63
NKX3.1
Wells:
P
CF
P
CF
P
CF
P = CaB34
CF = CaB34-CF
kDa
63
kDa
48
kDa
35
180
135
135
100
100
75
β-actin
AR
CDH1
*upper band from strong VIM signal remaining after strip & re-probe
P
CF
P
CF
kDa
63
kDa
48
100
35
75
63
VIM
KRT5
Figure 2A: Western blot data comparing protein expression between CaB34 and CaB34-CF.

## Slide 2
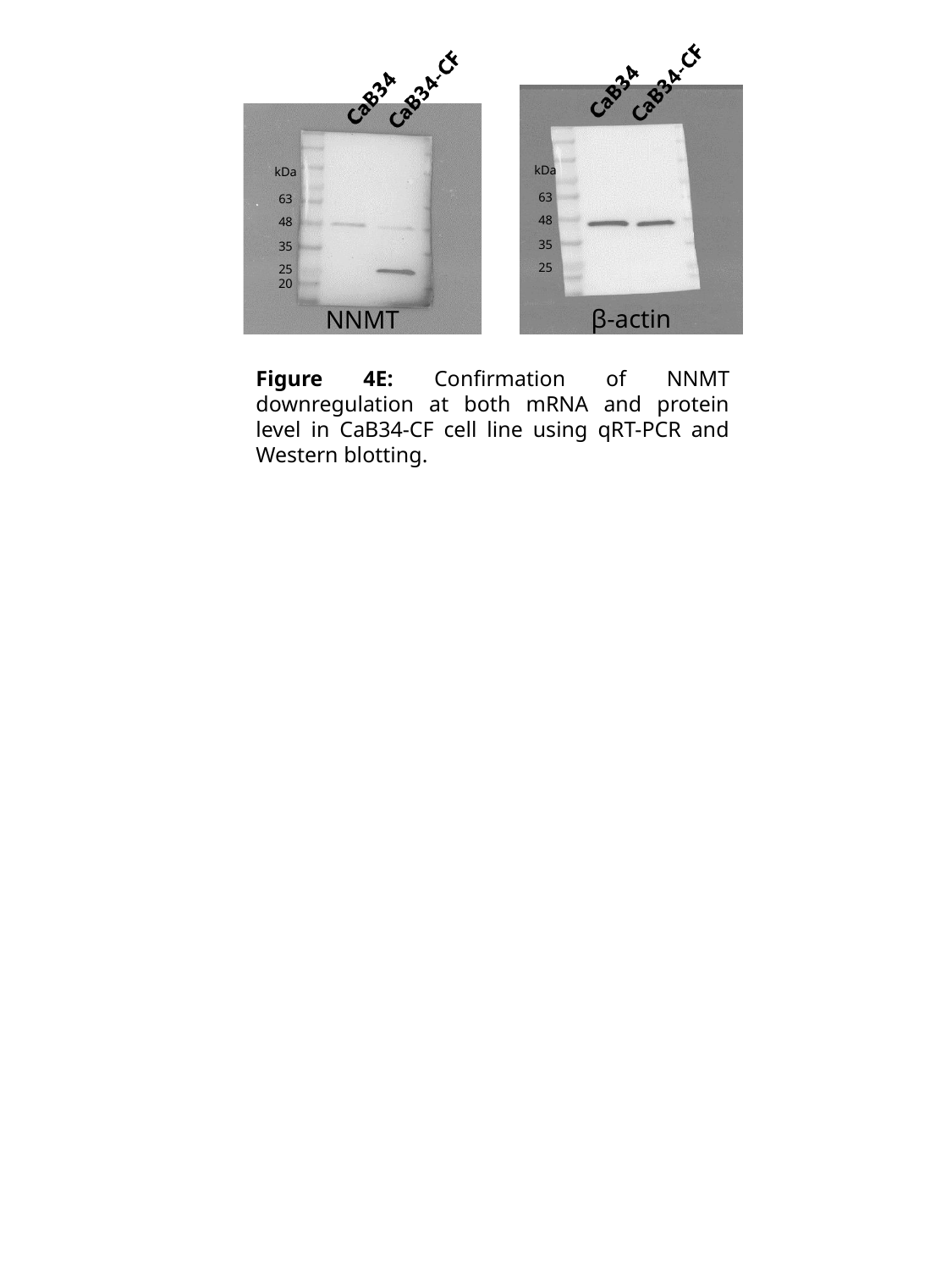

kDa
kDa
63
63
48
48
35
35
25
25
20
β-actin
NNMT
Figure 4E: Confirmation of NNMT downregulation at both mRNA and protein level in CaB34-CF cell line using qRT-PCR and Western blotting.

## Slide 3
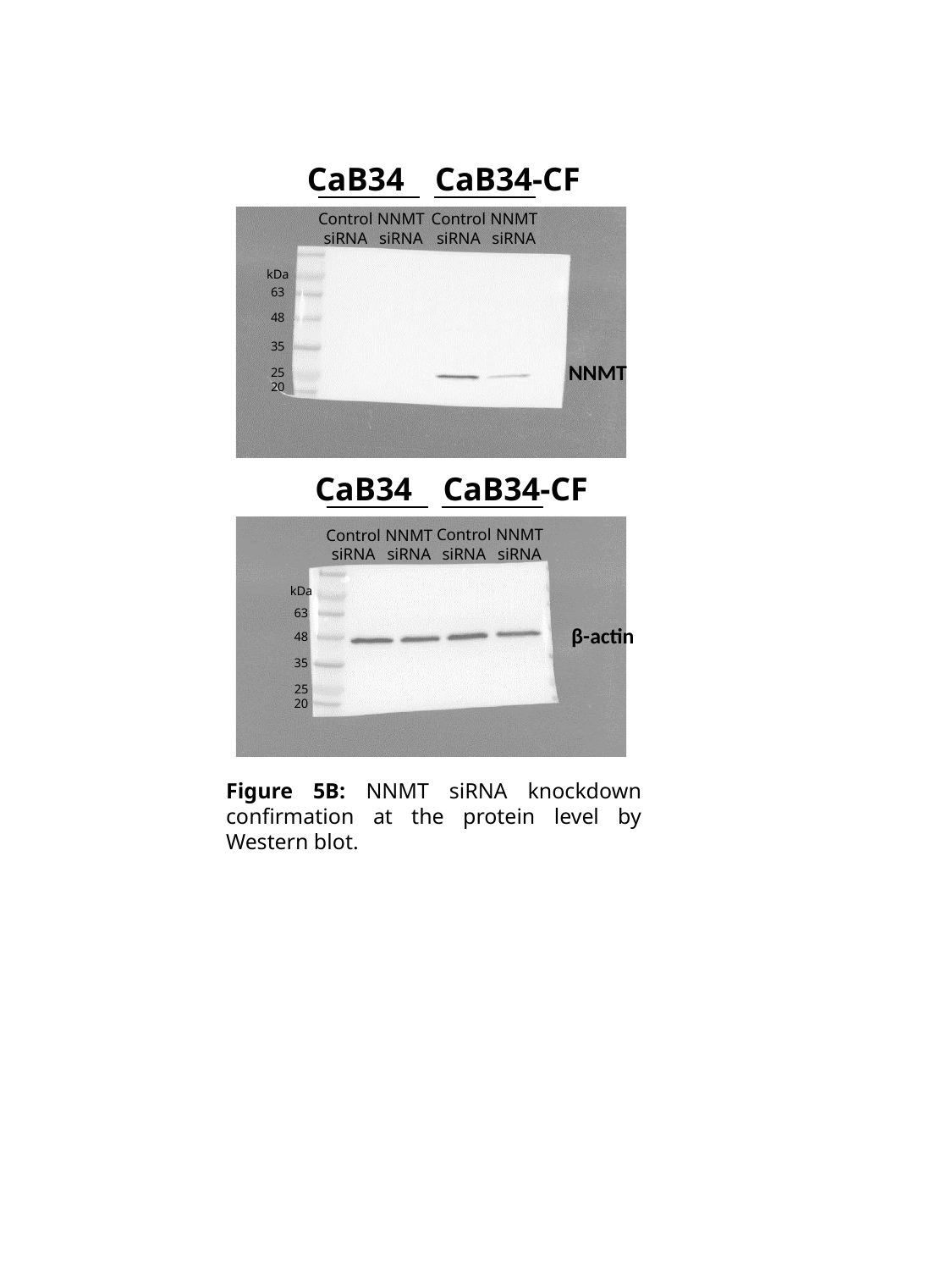

CaB34
CaB34-CF
Control
siRNA
NNMT
siRNA
Control
siRNA
NNMT
siRNA
kDa
63
48
35
NNMT
25
20
CaB34
CaB34-CF
Control
siRNA
NNMT
siRNA
Control
siRNA
NNMT
siRNA
kDa
63
β-actin
48
35
25
20
Figure 5B: NNMT siRNA knockdown confirmation at the protein level by Western blot.
