# Supplementary material for: A Novel Primary Cell Line Model of Localized Prostate Cancer and Radioresistance—A Role for Nicotinamide N-Methyltransferase
Source: Cells. 2025 May 31;14(11):819. doi: 10.3390/cells14110819 (PMC12153919; doi:10.3390/cells14110819)
Supplement: Supplementary file 1 [file cells-14-00819-s001.zip › Supplementary Table 1.pdf]

Antibody Target	Dilution	Vendor	Catalog no.	MW (kDa)
AMACR	1:1000	CST	3207	42
AR	1:1000	CST	5153	110
CDH1	1:1000	CST	3195	135
CHGA	1:1000	SantaCruz	sc-271738	80
KRT5	1:1000	CST	25807	62
KRT18	1:1000	Abcam	Ab32118	48
NKX3.1	1:1000	CST	83700	30
NNMT	1:500	Sigma	HPA059180	30
SYP	1:1000	CST	5461	38
TP63	1:1000	CST	39692	75
VIM	1:1000	CST	5741	57
B-actin	1:2000	CST	4967	45

Supplementary Table 1: Western blot antibodies. CST=Cell Signaling Technology. MW=molecular weight.
