# Supplementary material for: A Novel Primary Cell Line Model of Localized Prostate Cancer and Radioresistance—A Role for Nicotinamide N-Methyltransferase
Source: Cells. 2025 May 31;14(11):819. doi: 10.3390/cells14110819 (PMC12153919; doi:10.3390/cells14110819)
Supplement: Supplementary file 1 [file cells-14-00819-s001.zip › Supplementary Table 2.pdf]

| GENE SYMBOL | ASSAY ID |
|---|---|
| AR | Hs00171172_m1 |
| AMACR | Hs01091292_m1 |
| CDH1 | Hs01023894_m1 |
| CHGA | Hs00900375_m1 |
| KRT5 | Hs00361185_m1 |
| KRT8 | Hs01670053_m1 |
| NKX3.1 | Hs00171834_m1 |
| NNMT | Hs00196287_m1 |
| SYP | Hs00300531_m1 |
| TP63 | Hs00978340_m1 |
| VIM | Hs00185584_m1 |
| GAPDH | Hs99999905_m1 |

**Supplementary Table 2:** Taqman qRT-PCR primers.
