# Supplementary figures and images for: A Novel Primary Cell Line Model of Localized Prostate Cancer and Radioresistance—A Role for Nicotinamide N-Methyltransferase
Source: Cells. 2025 May 31;14(11):819. doi: 10.3390/cells14110819 (PMC12153919; doi:10.3390/cells14110819)

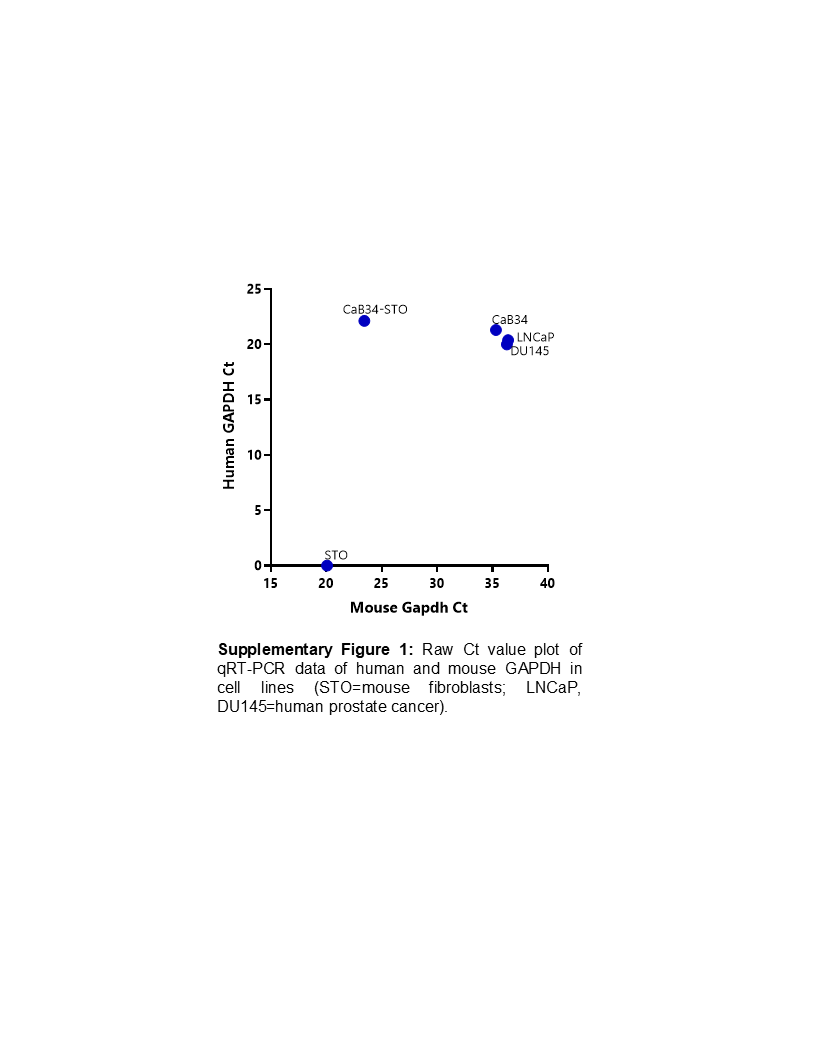

Supplement: Supplementary file 1 [file cells-14-00819-s001.zip › Figure S1.tif]

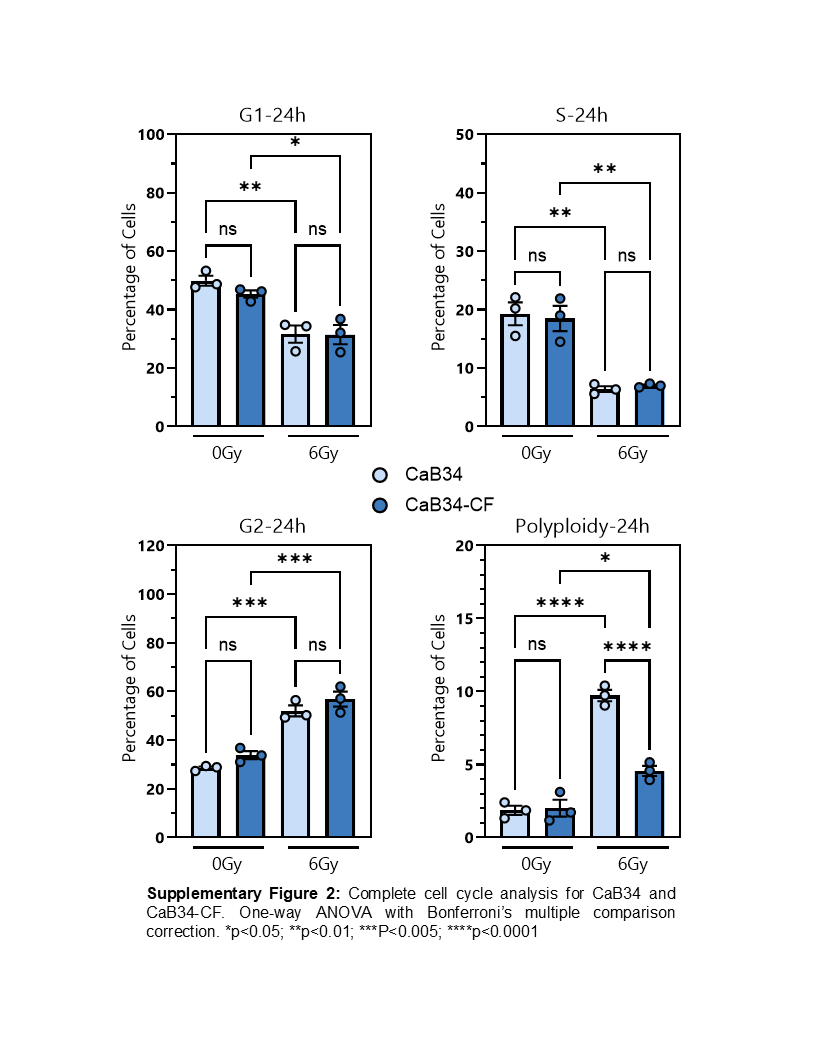

Supplement: Supplementary file 1 [file cells-14-00819-s001.zip › Figure S2.tif]

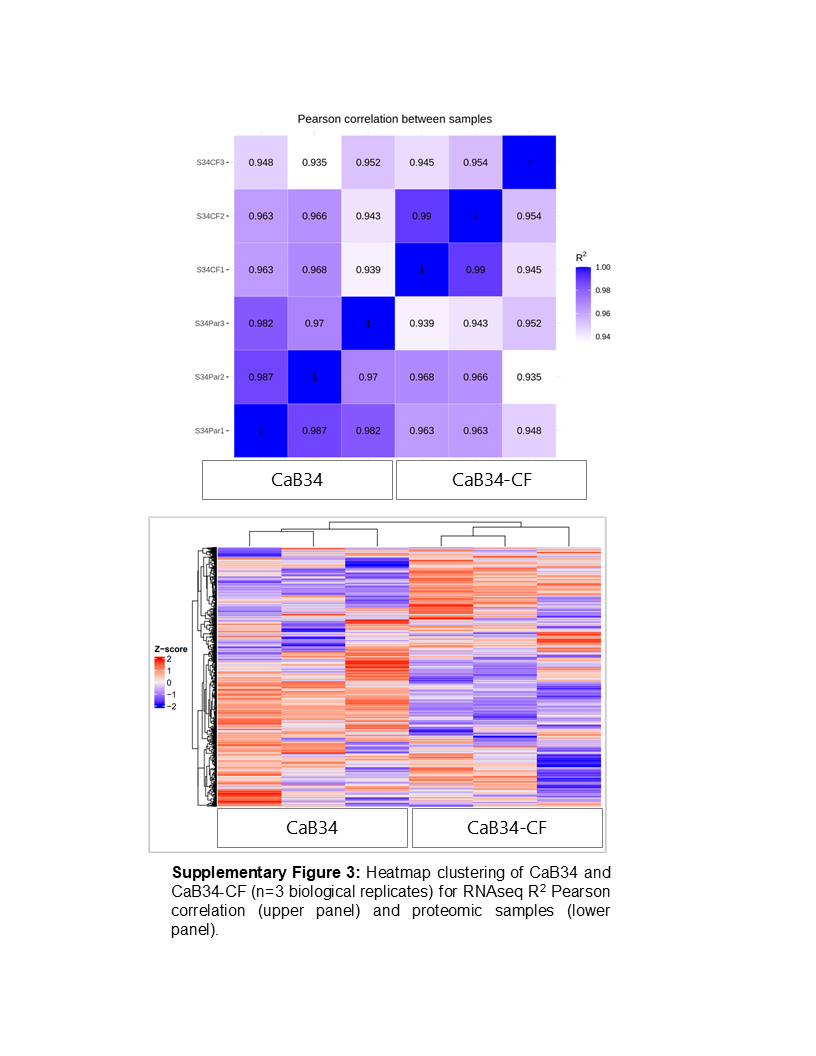

Supplement: Supplementary file 1 [file cells-14-00819-s001.zip › Figure S3.tif]
